# Ustisorbicillinols G and H, Two New Antibacterial Sorbicillinoids from the Albino Strain LN02 of Rice False Smut Fungus *Villosiclava virens*

**DOI:** 10.3390/molecules30143039

**Published:** 2025-07-20

**Authors:** Xuwen Hou, Mengyao Xue, Gan Gu, Dan Xu, Daowan Lai, Ligang Zhou

**Affiliations:** Department of Plant Pathology, College of Plant Protection, China Agricultural University, Beijing 100193, China; xwhou@cau.edu.cn (X.H.); xuemengyao2018@foxmail.com (M.X.); gugan@caas.cn (G.G.); cauxudan@cau.edu.cn (D.X.)

**Keywords:** rice false smut disease, *Villosiclava virens* (*Ustilaginoidea virens*), sorbicillinoids, ustisorbicillinols G and H, structural identification, antibacterial activity

## Abstract

*Villosiclava virens* (anamorph: *Ustilaginoidea virens*), the causal fungal pathogen of rice false smut, has been found to produce various secondary metabolites. The albino strain LN02 is a natural albino phenotype mutant of *V. virens* due to its inability to produce ustilaginoidins. The fermentation of *V. virens* LN02 was performed in solid rice medium to obtain fungal cultures, which were chemically investigated. After removing the known metabolites, two new dimeric sorbicillinoids, namely ustisorbicillinols G (**1**) and H (**2**), were isolated from the ethyl acetate extract. Their structures were elucidated using spectroscopic data analyses and quantum chemical calculations. Compounds **1** and **2** displayed antibacterial activity towards *Ralstonia solanacearum*, *Agrobacterium tumefaciens* and *Bacillus subtilis*, with median inhibitory concentration (IC_50_) values of 19.76–25.43 μg/mL for **1** and 25.35–45.48 μg/mL for **2**. The discovery of new sorbicillinoids will increase the diversity of the secondary metabolites of *V. virens* and provide candidates for the creation of new antimicrobials as well.

## 1. Introduction

Sorbicillinoids are a group of fungal hexaketide metabolites with a sorbyl side chain [1]. Based on their structural features and biosynthetic pathways, sorbicillinoids are grouped into monomeric, dimeric, trimeric and hybrid ones [2,3]. These sorbicillinoids show important physiological and ecological functions [4,5] and also have various types of biological activity, with potential pharmaceutical and agrochemical applications as antimicrobial, antioxidant, antivirus and anticancer agents [6,7,8,9,10].

Rice false smut (RFS) disease, caused by *Villosiclava virens* (anamorph: *Ustilaginoidea virens*), is a serious rice panicle disease in rice-producing areas around the world. In addition, the RFS pathogen *V. virens* can produce multiple secondary metabolites (SMs), some of which belong to mycotoxins and can be toxic to plants and animals, as well as posing a serious threat to the health of humans and domestic animals [11,12,13,14,15,16,17,18]. These toxic metabolites are involved in the pathogenic process of *V. virens* on rice plants, resulting in a decrease in the yield and quality of rice grains [19,20,21,22,23].

The albino strain LN02 of *V. virens* is a natural mutant with a white phenotype due to its inability to synthesize ustilaginoidins [24,25], which are the main SMs in the normal strain of *V. virens* [12,13,26,27]. Although the albino strain LN02 can produce sorbicillinoids like normal strains P1 and UV-8b [15,28], there are some differences in the types and relative content of sorbicillinoids between albino and normal strains, according to HPLC analysis [24].

**Figure 1:**
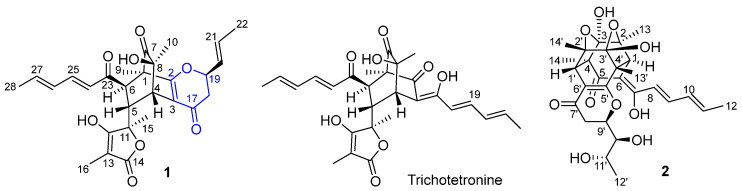
Structures of ustisorbicillinoids G (**1**) and H (**2**).

In the course of searching for new sorbicillinoids, the albino strain LN02 was cultured in solid rice medium, which led to the separation of two new dimeric sorbicillinoids, namely ustisorbicillinols G (**1**) and H (**2**) (Figure 1). Here, we report the isolation, structural characterization and antibacterial activity of these two compounds.

## 2. Results and Discussion

### 2.1. Structural Identification of Compounds ***1*** and ***2***

The EtOAc extract of fungi was successively subjected to repeated column chromatography over a normal-phase silica gel, reversed-phase silica gel (i.e., ODS) and Sephadex LH-20, as well as semi-preparative HPLC, to afford compounds **1** and **2** (Figure 1). The 1D and 2D NMR spectra, UV spectra and HRESIMS spectra of **1** and **2** are shown in Figures S3–S18.

Ustisorbicillinol G (**1**) was isolated as a yellow amorphous powder that exhibited a prominent pseudomolecular ion peak at *m*/*z* 495.2028 [M–H]^–^ (calcd for C_28_H_31_O_8_, 495.2024) in the HRESIMS spectrum (Figure S10), indicating a molecular formula of C_28_H_32_O_8_, with thirteen double-bond equivalents. Its UV spectrum indicated that it was a sorbicillinoid by analogy to the co-isolated trichotetronine (Figure 1).

A detailed analysis of the NMR data (Table 1) revealed that **1** was an analog of trichotetronine [29,30,31], as they showed similar NMR resonances, except for the lack of one olefinic double bond compared to the latter, while containing additionally one oxymethine group (C-19: *δ***_C_** 83.1; *δ***_H_** 4.92) and one methylene group (C-18: *δ***_C_** 42.1; *δ***_H_** 2.90, 2.43 each dd).

Further analysis of the 2D NMR spectra (Figure S1) allowed the establishment of the gross structure of **1**. In the ^1^H−^1^H COSY spectrum, correlations from Me-22 (*δ*_H_ 1.78, dd) to the olefinic H-21 (*δ*_H_ 5.95, dq), which correlated to another olefinic H-20 (*δ*_H_ 5.78, ddd), were seen. This proton (H-20) was correlated to the oxymethine (H-19), which in turn correlated to the methylene (H_2_-18), reflecting that one of the sorbyl chains in trichotetronine was modified in **1**. The HMBC correlations from H-9 (*δ*_H_ 1.02, s) to C-1 (*δ*_C_ 56.5), C-2 (*δ*_C_ 169.5) and C-7 (*δ*_C_ 209.0); from H-10 (*δ*_H_ 1.29, s) to C-4 (*δ*_C_ 41.2), C-7 and C-8 (*δ*_C_ 73.7); from H-4 (*δ*_H_ 3.57, d) to C-2, C-3 (*δ*_C_ 116.4), C-7 and C-8; from H-18a (*δ*_H_ 2.90, dd) to C-17 (*δ*_C_ 190.8) and C-19 (*δ*_C_ 83.1); and from H-4 to C-17, as well as from H-19 to C-2, suggested the presence of a dihydropyrone ring by linking C2 and C19 via an ether bond in the right part of the structure. Such a pyrone ring connecting the side chain and its cyclohexene ring was seen previously in ustisorbicillinols A–D from *U. virens* [28]. Hence, the planar structure of **1** was established (Figure 1).

The relative configuration of **1** was established to be the same as for trichotetronine, by comparing the *J* coupling constants and NOESY correlations, except for C-19. The relative configuration of C-19 was determined by comparing the coupling constants between H-19 and H_2_-18 (^3^*J*_H-19/H-18a_ and ^3^*J*_H-19/H-18b_: 13.8, 3.4 Hz) and the ^1^H/^13^C NMR data to those analogs that contained a dihydropyrone moiety [28].

**Figure 2:**
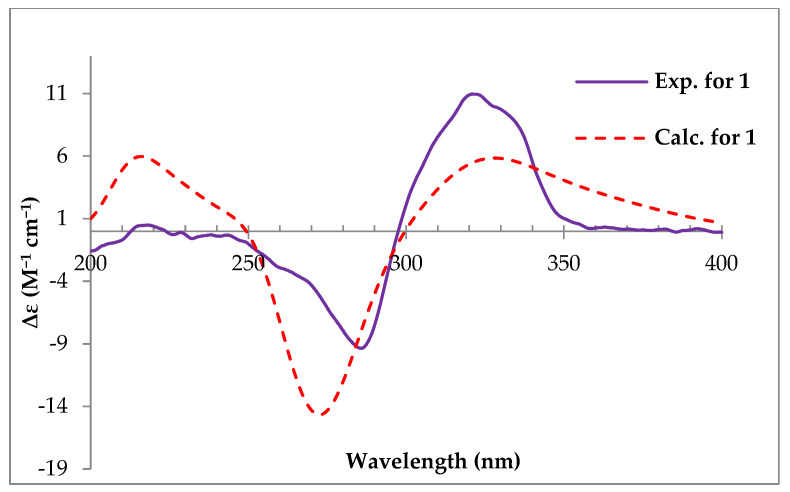
Calculated and experimental ECD spectra of **1**.

The absolute configuration of **1** was determined by ECD calculation with the calculated ECD curves of 1*R*, 4*S*, 5*S*, 6*R*, 8*S*, 11*R*, 19*R*-**1** at the PBE0/TZVP//B3LYP/6-31g(d) (IEFPCM, MeOH) level of theory, which matched the experimental ECD well (Figure 2). Therefore, compound **1** was elucidated and designated as ustisorbicillinol G (**1**).

The Michael addition of H_2_O to the C18/C19 double bond of trichotetronine could yield the corresponding 19-hydroxyl adduct, followed by the intramolecular nucleophilic attack of 19-OH to the keto group (C-2) to generate a semi-ketal, which should give rise to the dihydropyrone structure of **1** after dehydration.

Ustisorbicillinol H (**2**) was isolated as a yellow amorphous powder that had a prominent pseudomolecular ion peak at *m*/*z* 529.2088 [M−H]^−^ (calcd for C_28_H_33_O_10_, 529.2079) in the HRESIMS spectrum. The maximum UV absorptions at 200, 287 and 362 nm were similar to those of previously reported sorbicillinols [28,32].

Extensive analysis of the NMR data (Table 2) revealed that **2** was similar to dihydrotrichodimer ether A [32], except that the signals for one disubstituted double bond in dihydrotrichodimer ether A were replaced by two oxymethines (C11′: *δ*_C_ 67.6/ *δ*_H_ 3.69; C10′: *δ*_C_ 76.7/*δ*_H_ 3.64) in **2**. An analysis of the ^1^H−^1^H COSY and HMBC spectra revealed that the C-11′-C-10′ double bond was saturated in **2** and substituted with two hydroxyl groups. As in the COSY spectrum, Me-12′ (*δ*_H_ 1.12, d) was correlated to the oxymethine (CH-11′), which in turn was correlated to the second oxymethine (CH-10′), followed by its sequential correlation to the methine (CH-9′) of the pyrone ring. Key HMBC correlations from Me-12′ to C-11′/C-10′ unequivocally supported this assignment (Figure S2).

**Figure 3:**
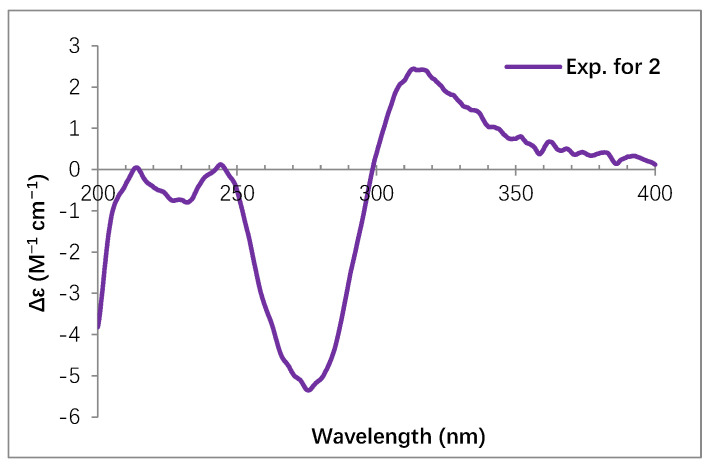
Experimental ECD spectrum of **2**.

**Figure 4:**
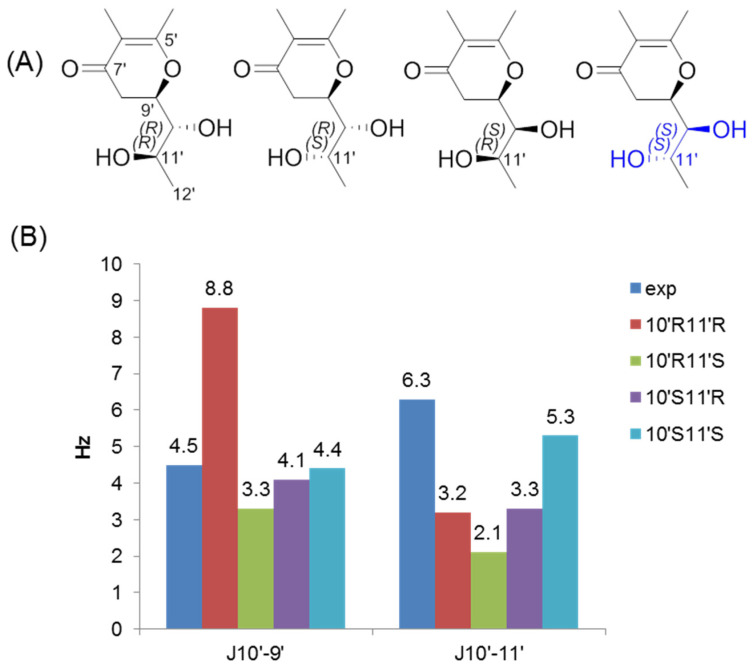
The experimental and calculated *J* values for **2**. (**A**) Four isomers with different C10′/C11′ stereochemistry were used for calculation. (**B**) A comparison of the *J* values of H10′/H9′ and H10′/H11′ between the experimental and computed structures at the mpw1pw91/6-311+g(2d,p) (PCM = acetone)// b3lyp/6-31g(d) level of theory.

The similar NOE correlations and coupling constants (^3^*J*_H-H_) to dihydrotrichodimer ether A indicated similar relative stereochemistry in **2**, except for C-11′/C-10′. In addition, the CD spectrum of **2** displayed cotton effects at 317, 278, 246, 232 and 216 nm (Figure 3), which were similar to those of dihydrotrichodimer ether A [32], implying the same absolute configuration for the cage structure, except for C-10′ and C-11′. Their configurations were determined by the analysis of the experimental and calculated *J* values for the model structures (Figure 4). A truncated structure of **2** was used for the calculations, in which both the C5′ and C6′ substituents were replaced by a methyl group. Since the absolute configuration of C9′ has been determined as stated above, only four isomers differing at C-10′/C-11′ were considered (Figure 4A). As shown in Figure 4B, the calculated values for the 10′*S*,11′*S*-isomer fitted well with the experimental data. Hence, an *S* configuration was assigned for both C10′ and C11′. It is possible that the olefinic bond at C10′/C11′ of dihydrotrichodimer ether A was first epoxidized, and then the epoxide ring opened by the nucleophilic attack of water should explain the biosynthesis of **2**.

### 2.2. Antibacterial Activity of Compounds ***1*** and ***2***

Compounds **1** and **2** were examined for their antibacterial activity and cytotoxicity in six human cancer cell lines. The positive controls for the antibacterial and cytotoxic activity evaluation were streptomycin sulfate and Taxol, respectively. Compound **1** showed significant antibacterial activity against *Ralstonia solanacearum*, *Agrobacterium tumefaciens* and *Bacillus subtilis*, with IC_50_ values of 24.33, 19.76 and 25.43 μg/mL, respectively. Compound **2** also had lower or similar antibacterial activity against *R. solanacearum*, *A. tumefaciens* and *B. subtilis*, with IC_50_ values of 35.42, 45.48 and 25.35 μg/mL, respectively (Table 3). The sorbicillinoids, including ustisorbicillinol B, dihydrotrichodimer ether A, oxosorbicillinol, bisvertinolone and demethyltrichodimerol, isolated from the normal RFS strain UV8b also showed obvious antibacterial activity [28]. This indicates that these sorbicillinoids have protective effects in RFS fungi (*V. virens*) against bacteria. The ecological significance of sorbicillinoids in *V. virens* against bacteria need to be further verified and investigated.

Compounds **1** and **2** were also examined for their cytotoxic activity. However, both compounds did not show any cytotoxic activity (Table S1).

Fungi, such as marine fungi [33,34], endophytic fungi [35,36] and pathogenic fungi [37,38], can produce large amounts of SMs, which have been considered a treasure trove of novel bioactive compounds [39].

The potential biosynthetic gene clusters (BGCs) for SMs in *V. virens* were analyzed by the method of antiSMASH. Nineteen gene clusters for SMs were identified, among which the polyketide BGCs [5] and ribosomal peptide BGCs [40] were well expressed. Many SMs have been identified in *V. virens*. They mainly include polyketides and cyclopeptides such as ustilaginoidins [12,13], ustiloxins [11,14] and sorbicillinoids [15,28]. In addition to their functions in the development, stress responses and pathogenicity of *V. virens* [5], these SMs show various types of biological activity, such as cytotoxic, antimicrobial, phytotoxic and antioxidant activity [12,13,14]. Therefore, *V. virens* has been considered an important SM-producing fungus.

The bioinformatics analysis demonstrated that the number of BGCs encoding SMs in the *V. virens* genome was much larger than the number of identified SMs, revealing that there were plenty of silent gene clusters in this fungus. In order to either reveal additional quantities of SMs or increase their production in *V. virens*, the metabolic regulation [41,42,43,44,45,46], metabolic shunting [47,48,49,50,51] or heterologous expression of BGCs [52] and promoter replacement [53,54] for the activation of fungal silent BGCs to mine the chemical diversity of this fungus might be effective strategies.

In this study, the discovery of two new dimeric sorbicillinoids with their antibacterial activity from the albino strain LN02 increased the diversity of SMs in *V. virens*. Many sorbicillinoids have been screened to show antibacterial and antifungal activity [2,3,7]. Some sorbicillinoids, such as ustisorbicillinol B, dihydrotrichodimer ether A, oxosorbicillinol, bisvertinolone and demethyltrichodimerol, previously isolated from *V. virens*, have also been screened to show antibacterial activity [28]. These sorbicillinoids might play important roles between *V. virens* and its surrounding microorganisms.

Some sorbicillinoids, such as trichotetronine (also named bislongiquinolide), have been screened to show cytotoxic activity [20]. Unfortunately, the trichotetronine analogs ustisorbicillinoids G (**1**) and H (**2**) reported in this study have not been shown to have cytotoxic activity (Table S1). Possible reasons might be the differences in their structures, the cell lines employed and the experimental conditions between them. The cytotoxic activity of ustisorbicillinoids G (**1**) and H (**2**) needs further investigation.

The albino strain LN02 was proven as the natural deletion mutant of ustilaginoidin biosynthesis, which led to the easy recognition and isolation of sorbicillinoids **1** and **2**. It is possible that the absence of ustilaginoidins, which were the main metabolites in the normal strain, led to the trace metabolites being detectable in the albino strain. This approach, based on metabolic shunting through genetic dereplication, has been previously reported to discover new SMs (especially trace metabolites) from other fungal species, eliminating the production of the main metabolites and enabling minor metabolites to be detectable and separable [47,48,49,50,51].

## 3. Materials and Methods

### 3.1. Fungus and Fermentation

The albino strain LN02 of rice false smut fungus *V. virens* was stored in a refrigerator at −80 °C before use [24]. Before strain LN02 was cultured on potato dextrose agar medium (200 g/L of potato, 20 g/L of dextrose and 20 g/L of agar, PDA) at 28 °C for 14 days, a few agar plugs (0.3 cm × 0.3 cm) containing mycelia were added into a 250 mL Erlenmeyer flask containing 100 mL of potato dextrose liquid medium, which was PDA without agar. The liquid culture was incubated in a rotatory shaker for 5 days at 180 rpm under 28 °C to produce the seed culture, which was used to inoculate the solid rice medium (1000 mL Erlenmeyer flask containing 100 g rice and 110 mL water). The fermentation was carried out using a total of 10 kg rice at room temperature (RT) under static conditions in darkness for two months.

### 3.2. Extraction and Separation

The rice cultures, which had been cultivated for two months, were combined, dried and pulverized. The dry materials were extracted with ethyl acetate (EtOAc) three times at room temperature, each for 5 days, which led to 80 g of EtOAc extract after removing the solvent. Then, the extract was subjected to vacuum liquid chromatography (VLC) over silica gel (i.d. 8 cm × 40 cm) by eluting it with a different mixture of petroleum ether (Shanghai Tichem Chemical Co., Ltd., Shanghai, China) and dichloromethane (Shanghai Tichem Chemical Co., Ltd., Shanghai, China) (PE/CH_2_Cl_2_), followed by CH_2_Cl_2_/EtOAc, EtOAc/MeOH. Fractions were pooled according to TLC analysis, and 14 fractions were obtained (Frs. A–M). Fr. I (5.0 g) was subjected to gel permeation chromatography over a Sephadex LH-20, eluting with CH_2_Cl_2_/MeOH (1:1, *v*/*v*) to obtain 12 subfractions. Compound **1** (4.5 mg) was purified from subfr. I-3 by semi-preparative HPLC, eluting with 65% MeOH in H_2_O. Fr. M was chromatographed over RP-18 to afford 25 subfractions. Subfr. M-23 was subjected to gel permeation chromatography over a Sephadex LH-20, eluting with CH_2_Cl_2_/MeOH (1:1, *v*/*v*) to obtain 14 fractions. Compound **2** (5.5 mg) was further purified from the fifth fraction by semi-preparative HPLC, eluting with 65% MeOH in H_2_O.

Ustisorbicillinol G (**1**): yellow amorphous powder; [α]^24^_D_ +7.33 (c 0.20, MeOH); ECD (*c* = 0.60 mM, MeOH) *λ* 216, 258, 286, 319 nm; UV (MeOH) *λ*_max_ 200, 228, 290 nm; ^1^H NMR (CD_3_OD, 500 MHz), ^13^C NMR (CD_3_OD, 125 MHz) see Table 1; HRESIMS *m*/*z* 495.2028 [M−H]^–^ (calcd for C_28_H_31_O_8_, 495.2024).

Ustisorbicillinol H (**2**): yellow amorphous powder; [*α*]^24^_D_ +9.67 (*c* 0.20, MeOH); ECD (*c* = 0.57 mM, MeOH) *λ* 216, 232, 246, 278, 317 nm; UV (MeOH) *λ*_max_ 200, 287, 362 nm; ^1^H NMR (CD_3_COCD_3_, 500 MHz), ^13^C NMR (CD_3_COCD_3_, 125 MHz) see Table 2; HRESIMS *m*/*z* 529.2088 [M−H]^–^ (calcd for C_28_H_33_O_10_, 529.2079).

### 3.3. Calculation of ECD

The conformers of compound **1** were generated by Spartan 14 (v1.1.4) using the MMFF94 molecular mechanics force field calculation with 3.0 kcal/mol cutoff energy [55]. The software package Gaussian 09 (E.01) was used to perform DFT calculations. The optimization and frequency calculation of conformers were performed at the B3LYP/6-31G(d) level. The theoretical ECD (TDDFT) of compound **1** was calculated at the PBE0/TZVP level with the IEF-PCM solvent (MeOH) model as well. SpecDis v1.70.1 was used to simulate the ECD curve, with σ/γ value 0.3 eV [56]. The calculated ECD curve of each conformer was Boltzmann-averaged based on their Gibbs free energy. The calculated ECD spectra were UV-shifted by +10 nm for comparison with the measured spectrum. The nuclear spin–spin coupling *J* (Hz) for the truncated models of **2** was calculated at the mpw1pw91/6-311+g(2d,p) (PCM = acetone) level.

### 3.4. Antibacterial Assay

The antibacterial activity of compounds **1** and **2** was evaluated on three bacterial strains, namely *Ralstonia solanacearum* (*R. solanacearum*), *Agrobacterium tumefaciens* (*A. tumefaciens*) and *Bacillus subtilis* (*B. subtilis*), which were kindly provided by the Plant Pathology Department of the Plant Protection College, China Agricultural University (CAU). Streptomycin sulfate (Sigma, Shanghai, China) with purity greater than 95% was used as the positive control. Dimethyl sulfoxide (DMSO) for dissolving compounds was used as the blank control. Both the minimum inhibitory concentration (MIC) values and median inhibitory concentration (IC_50_) values of the compounds and the positive control were obtained in sterile microplates with 96 wells by the modified broth dilution test, as described previously [28].

### 3.5. Cytotoxic Activity Assay

The cytotoxicity of the compounds was evaluated on human carcinoma cells using the microculture methyl thiazolyl tetrazolium (MTT) assay, as described previously [12]. The tested human cell lines included human colon cancer (HCT116), human breast cancer (MDA-MB-231), human gastric cancer (BGC823), human hepatoma (Huh-7), human non-small cell lung cancer (PC9) and human pancreatic cancer (PANC-1) cell lines, which were provided by the Institute of Materia Medica, Chinese Academy of Medical Sciences. The positive control was Taxol. DMSO, which was used to dilute the compounds, served as the blank control.

## 4. Conclusions

In summary, two novel dimeric sorbicillinoids, namely ustisorbicillinols G (**1**) and H (**2**), were isolated from albino strain LN02 cultures of rice false smut fungus *V. virens*. The structures of **1** and **2** were elucidated by comprehensive spectroscopic analysis, together with quantum-chemical calculations. Both compounds showed antibacterial activity and could represent candidates for the creation of new antimicrobials. Furthermore, the discovery of new sorbicillinoids will increase the diversity of the secondary metabolites of *V. virens.* In order to increase the production of ustisorbicillinols G (**1**) and H (**2**), it is necessary to optimize the culture conditions to enhance their biosynthesis. In addition, the biosynthesis regulation mechanisms and the physiological and ecological functions of **1** and **2** in *V. virens*, as well as their potential application as antimicrobial agents, need further investigation.

## Figures and Tables

**Table 1:** ^1^H (500 MHz) and ^13^C (125 MHz) NMR data of **1** (CD_3_OD)

Position	*δ*_C_, Type	*δ*_H_, Mult. (*J* in Hz)
1	56.5, C	
2	169.5, C *	
3	116.4, C	
4	41.2, CH	3.57, d (2.0)
5	44.5, CH	3.03, dd (6.1, 2.0)
6	53.8, CH	2.79, d (6.1)
7	209.0, C *	
8	73.7, C	
9	11.2, CH_3_	1.02, s
10	25.2, CH_3_	1.29, s
11	85.1, C	
12	181.6, C *	
13	95.5, C *	
14	177.9, C *	
15	22.0, CH_3_	1.45, s
16	6.3, CH_3_	1.56, s
17	190.8, C	
18	42.1, CH_2_	2.90, dd (16.7, 13.8); 2.43, dd (16.7, 3.4)
19	83.1, CH	4.92, m
20	129.1, CH	5.78, ddd (15.4, 7.4, 1.8)
21	132.9, CH	5.95, dq (15.4, 6.4)
22	17.9, CH_3_	1.78, dd (6.4, 1.8)
23	203.2, C *	
24	128.9, CH	6.10, d (15.5)
25	147.9, CH *	7.14, dd (15.5, 10.8)
26	131.8, CH	6.31, dd (15.1, 10.8)
27	145.1, CH	6.41,dq (15.1, 6.8)
28	19.1, CH_3_	1.90, d (6.8)

* The signals were further confirmed through HMBC spectra.

**Table 2:** ^1^H (500 MHz) and ^13^C (125 MHz) NMR data of **2** (CD_3_COCD_3_)

Position	*δ*_C_, Type	*δ*_H_, Mult. (*J* in Hz)
1	59.0, CH	3.11, d (2.4)
2	79.6, C	
3	105.3, C	
4	60.8, C	
5	202.3, C	
6	105.5, C	
7	174.3, C	
8	120.5, CH	6.52, dd (14.8, 4.2)
9	143.1, CH	7.30, ddd (14.8, 11.0, 7.0)
10	132.1, CH	6.42, m
11	139.8, CH	6.26, dqd (15.0, 7.6, 3.8)
12	19.0, CH_3_	1.88, d (7.6)
13	22.2, CH_3_	1.34, s
14	19.9, CH_3_	1.32, s
1′	53.9, CH	3.19, s
2′	79.4, C	
3′	105.0, C	
4′	56.4, C	
5′	173.7, C	
6′	109.5, C	
7′	189.8, C	
8′	36.6, CH_2_	2.66, dd (16.9, 14.6); 2.37, dd (16.9, 3.2)
9′	81.6, CH	4.15, ddd (14.6, 4.43.2)
10′	76.7, CH	3.64, dd (6.3, 4.5)
11′	67.6, CH	3.69, dq (6.3, 6.1)
12′	19.5, CH_3_	1.12, d (6.1)
13′	19.5, CH_3_	1.41, s
14′	21.7, CH_3_	1.24, s

**Table 3:** Antibacterial activity of compounds **1** and **2**.

Bacterium	MIC/IC_50_ (μg/mL)	Compound		
		1	2	CK^+^
*R. solanacearum*	MIC	32.00	64.00	2.50
	IC_50_	24.33 ± 2.04	35.42 ± 1.29	1.15 ± 0.27
*A. tumefaciens*	MIC	32.00	64.00	5.00
	IC_50_	19.76 ± 2.77	45.48 ± 3.22	1.12 ± 0.17
*B. subtilis*	MIC	32.00	32.00	5.00
	IC_50_	25.43 ± 2.74	25.35 ± 3.98	1.37 ± 0.51

Note: The positive control was streptomycin sulfate. MIC, minimum inhibitory concentration; IC_50_, median inhibitory concentration.

## Data Availability

The data are contained within the article or Supplementary Materials.

## Supplementary Materials

The following supporting information can be downloaded at: https://www.mdpi.com/article/10.3390/molecules30143039/s1. General experimental procedures; Figure S1: Key 2D NMR correlations of **1**; Figure S2: Key 2D NMR correlations of **2**; Figure S3: ^1^H NMR spectrum of **1** (CD_3_OD, 500 MHz); Figure S4: ^13^C NMR spectrum of **1** (CD_3_OD, 125 MHz); Figure S5: HSQC spectrum of **1**; Figure S6: ^1^H–^1^H COSY spectrum of **1**; Figure S7: HMBC spectrum of **1**; Figure S8: NOESY spectrum of **1**; Figure S9: UV spectrum of **1** (100% MeOH/H_2_O, extracted from HPLC-DAD data); Figure S10: HRESIMS spectrum of **1**; Figure S11: ^1^H NMR spectrum of **2** (CD_3_COCD_3_, 500 MHz); Figure S12: ^13^C NMR spectrum of **2** (CD_3_COCD_3_, 125 MHz); Figure S13: HSQC spectrum of **2**; Figure S14: ^1^H–^1^H COSY spectrum of **2**; Figure S15: HMBC spectrum of **2**; Figure S16: NOESY spectrum of **2**; Figure S17: UV spectrum of **2** (100% MeOH/H2O, extracted from HPLC-DAD data); Figure S18: HRESIMS spectrum of **2**; Table S1: Cytotoxic activity of compounds **1** and **2**.
