# Supplementary material for: Ustisorbicillinols G and H, Two New Antibacterial Sorbicillinoids from the Albino Strain LN02 of Rice False Smut Fungus Villosiclava virens
Source: Molecules. 2025 Jul 20;30(14):3039. doi: 10.3390/molecules30143039 (PMC12300444; doi:10.3390/molecules30143039)
Supplement: Supplementary file 1 [file molecules-30-03039-s001.zip › molecules-3728147-supplementary.pdf]

Supplementary Materials:

Ustisorbicillinols G and H, Two New Antibacterial Sorbicillinoids from the Albino Strain LN02 of Rice False Smut Fungus *Villosiclava virens*

Xuwen Hou †, Mengyao Xue †, Gan Gu †, Dan Xu, Daowan Lai * and Ligang Zhou *

Department of Plant Pathology, College of Plant Protection, China Agricultural University, Beijing 100193, China; xwhou@cau.edu.cn (X.H.); xuemengyao2018@foxmail.com (M.X.); gugan@caas.cn (G.G.); cauxudan@cau.edu.cn (D.X.)
* Correspondence: dwlai@cau.edu.cn (D.L.); lgzhou@cau.edu.cn (L.Z.)
† These authors contributed equally to this work.

General Experimental Procedures

The UV spectra were recorded on a TU-1810 UV−vis spectrophotometer (Beijing Persee General Instrument Co., Ltd., Beijing, China). The spectra of circular dichroism (CD) were recorded on a JASCO J-1500 CD spectrometer (JASCO Corp., Tokyo, Japan). Specific rotations of optical rotatory dispersion (ORD) were recorded on a Rudolph Autopol IV automatic polarimeter (Rudolph Research Analytical, NJ, USA). The spectra of high-resolution electrospray ionization mass spectrometry (HRESIMS) were recorded on an LC 1260-Q-TOF/MS 6520 machine (Agilent Technologies, CA, USA).

^{1}H, ^{13}C, and 2D NMR (HMBC, HSQC, ^{1}H–^{1}H COSY, NOESY) spectra were measured on an Avance 500 NMR spectrometer (Bruker BioSpin, Zürich, Switzerland). Chemical shifts were expressed in δ (ppm) referring to the solvent residual peaks at δ_H 3.31, δ_C 49.0 for CD_3OD, δ_H 2.05, δ_C 206.3 for CD_3COCD_3, and coupling constants (*J*) were in hertz.

The column chromatography (CC) was performed on the normal-phase silica gel (100–200 mesh, Qingdao Marine Chemical Co. Ltd., Qingdao, China), reversed-phase (RP)-C18 gel (20–45 μm, Fuji Silysia Chemical Ltd., Japan), and Sephadex LH-20 (40–70 μm; Amersham Pharmacia Biotech, Uppsala, Sweden).

The HPLC–DAD analysis was equiped with a Shimadzu LC-20A instrument containing an SPD-M20A photodiode array detector (Shimadzu Corp., Tokyo, Japan) and an analytic C_{18} HPLC column (250 mm × 4.6 mm i.d., 5 μm; Phenomenex Inc., Torrance, CA, USA). Semi-preparative HPLC separation was carried out on a Lumtech instrument (Lumiere Tech. Ltd., Beijing, China) equipped with a K-501 pump (flow rate: 3 mL/min) and a K-2501 UV detector using a Luna-C_{18} column (250 mm × 10 mm i.d., 5 μm, Phenomenex Inc., USA).

(**A**)

(**B**)

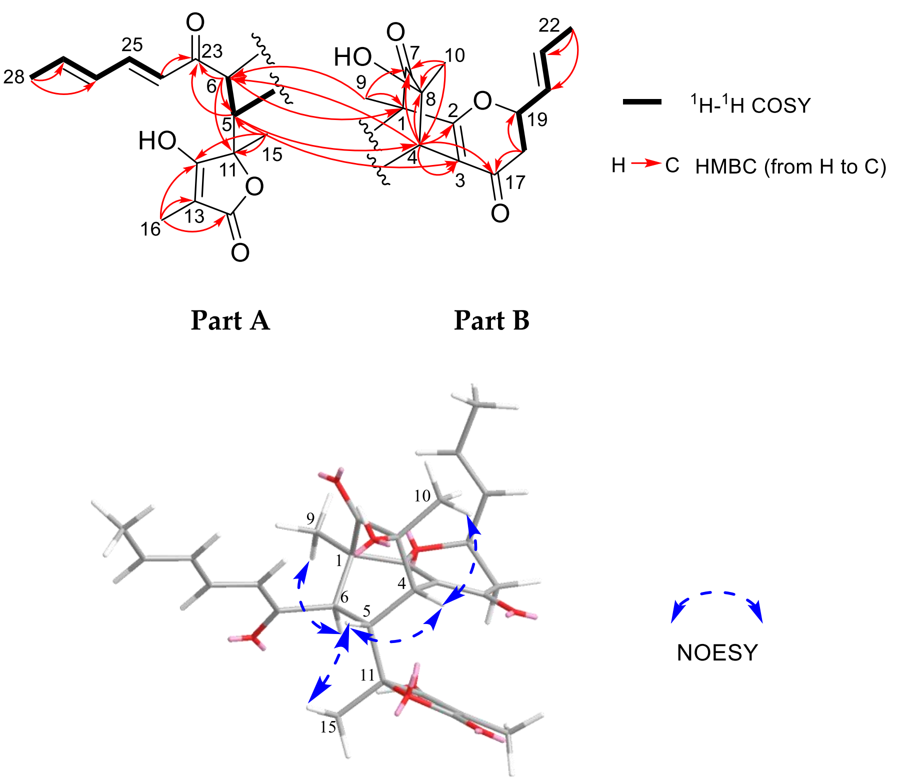

Figure S1. Key 2D NMR correlations of **1**. (**A**) The key correlations of ^{1}H–^{1}H COSY and HMBC. (**B**) The key correlations of NOESY.

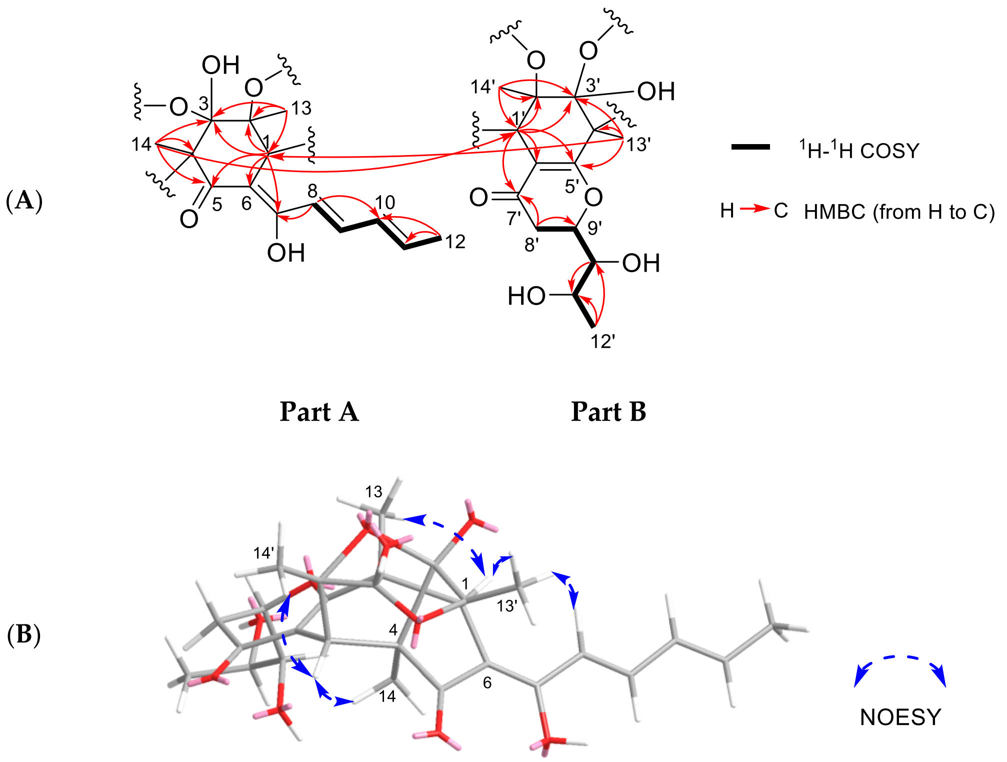

Figure S2. Key 2D NMR correlations of **2**. (**A**) The key correlations of ^{1}H–^{1}H COSY and HMBC. (**B**) The key correlations of NOESY.

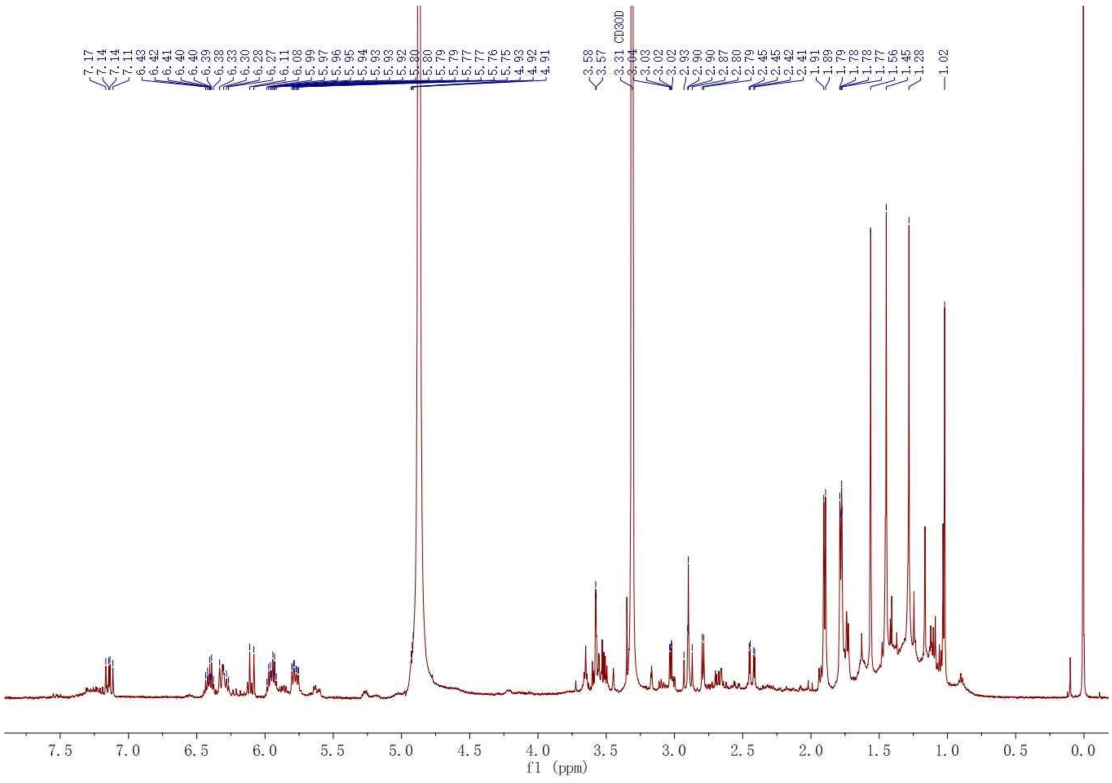

Figure S3. ^{1}H NMR spectrum of **1** (CD_3OD, 500 MHz).

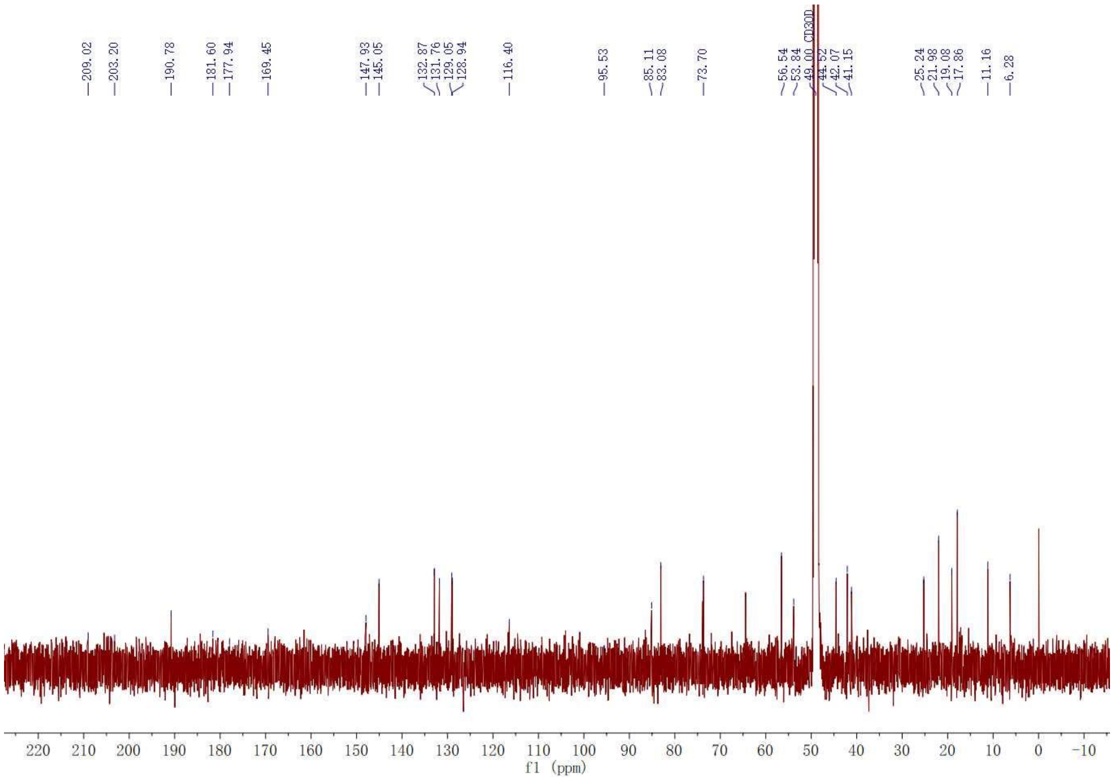

Figure S4. ^{13}C NMR spectrum of **1** (CD_3OD, 125 MHz).

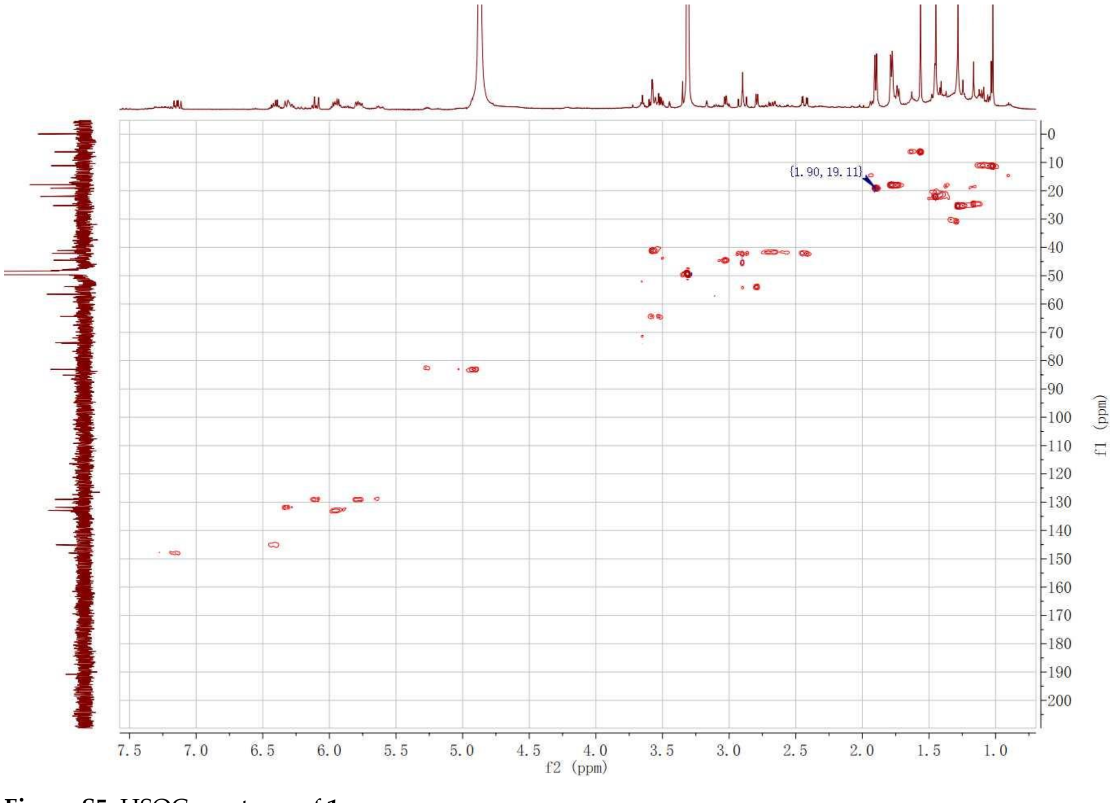

Figure S5. HSQC spectrum of **1**.

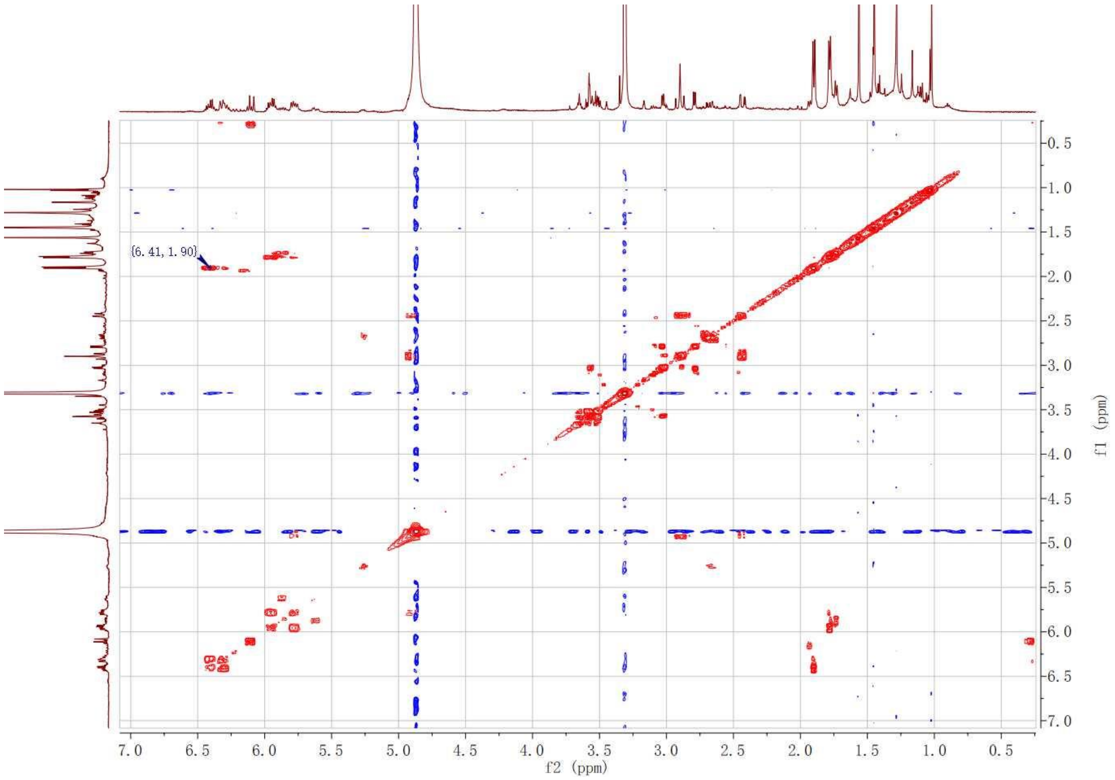

Figure S6. ^{1}H–^{1}H COSY spectrum of **1**.

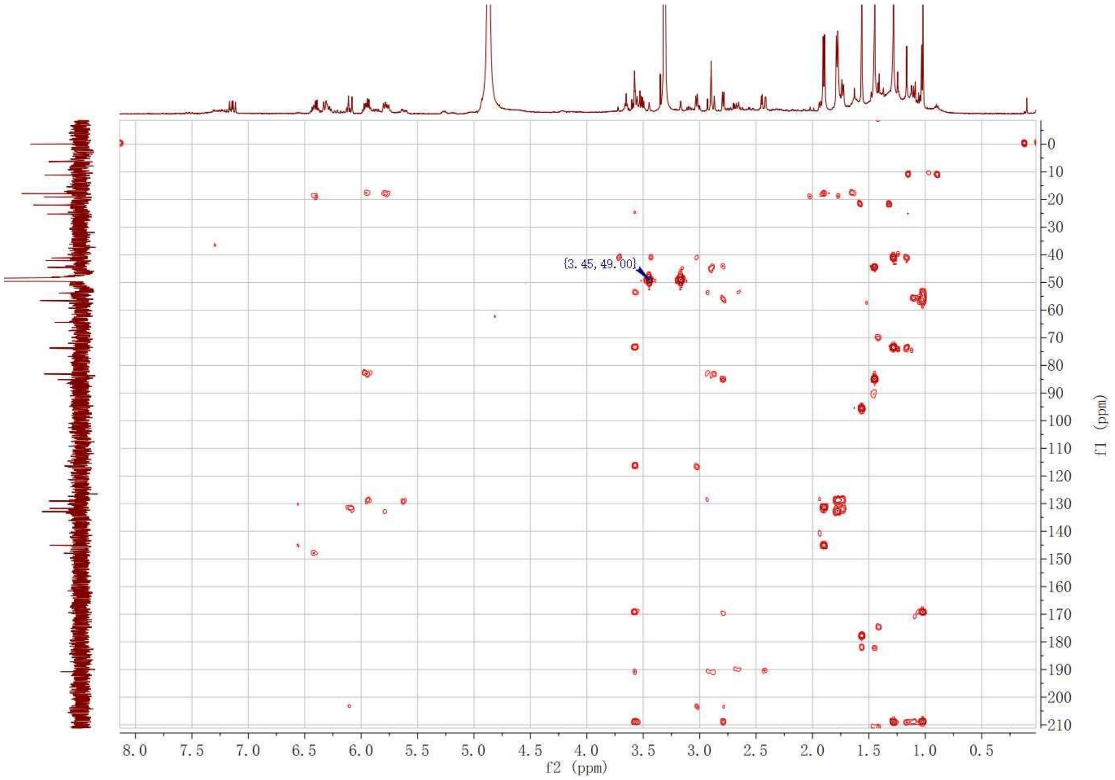

Figure S7. HMBC spectrum of **1**.

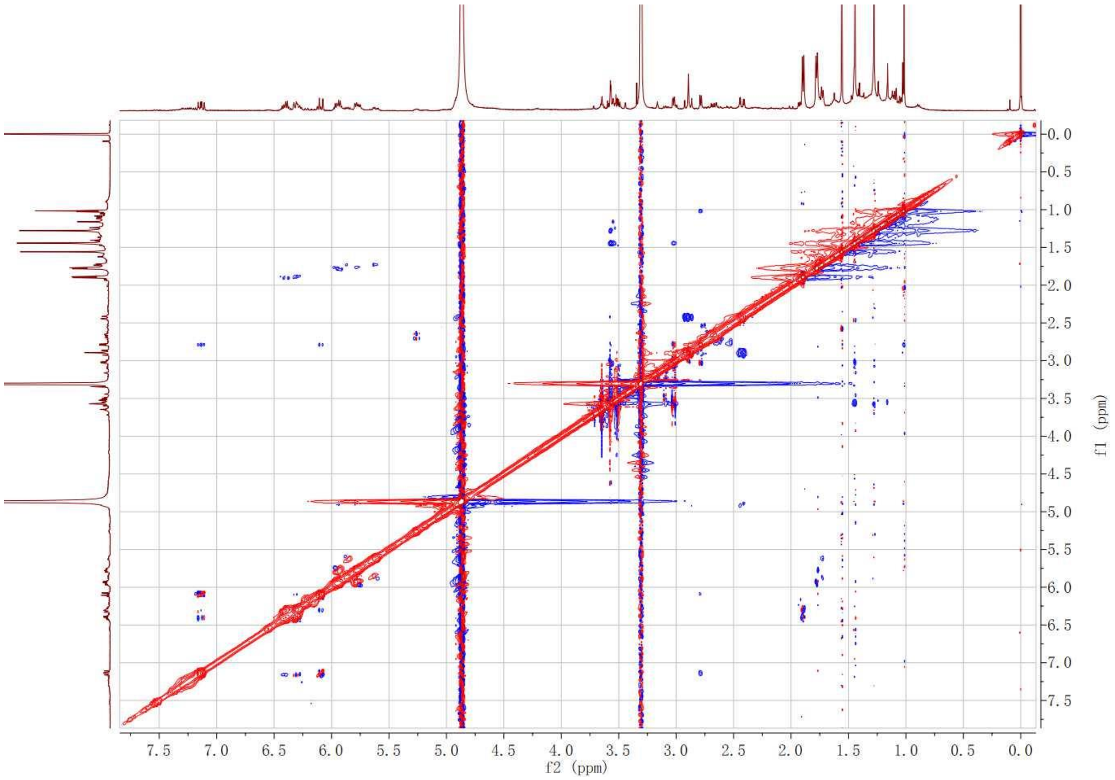

Figure S8. NOESY spectrum of **1**.

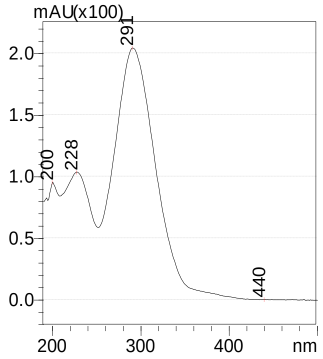

Figure S9. UV spectrum of **1** (100% MeOH/H_2O, extracted from HPLC-DAD data).

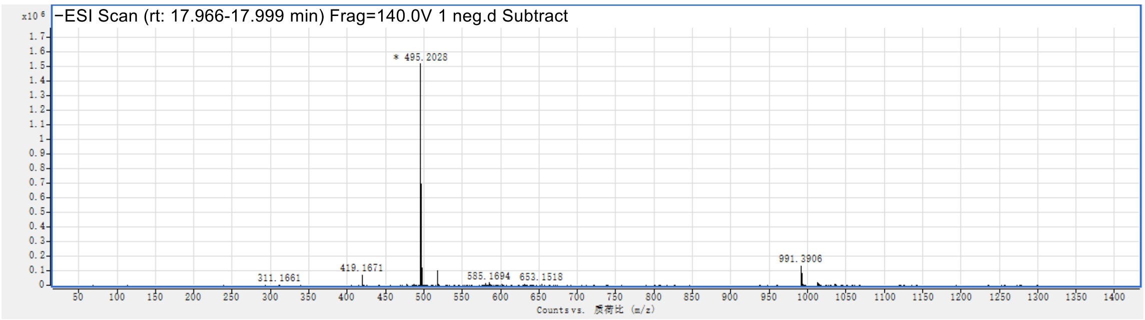

m/z	Calc *m/z*	Diff(ppm)	z	Abund	Formula	Ion
495.2028	495.2024	3.29	-1	1518756	$C_{28}H_{31}O_8$	$[M - H]^-$

Figure S10. HRESIMS spectrum of **1**.

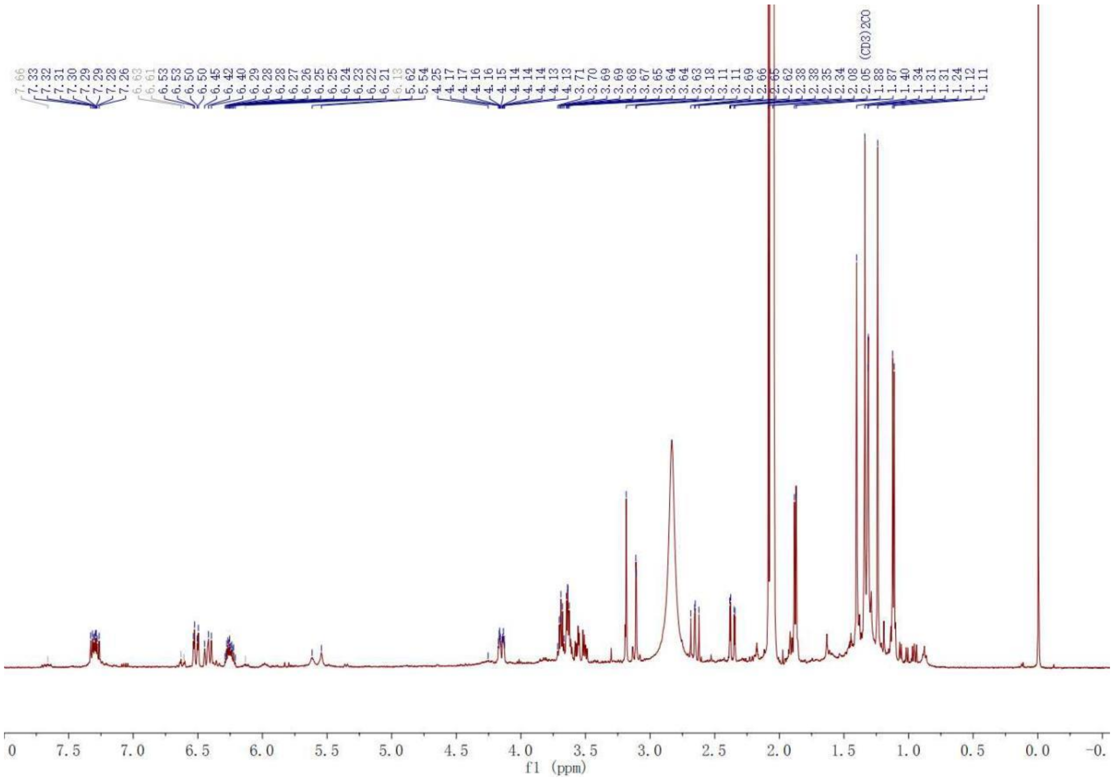

Figure S11. ^{1}H NMR spectrum of **2** (CD_3COCD_3, 500 MHz).

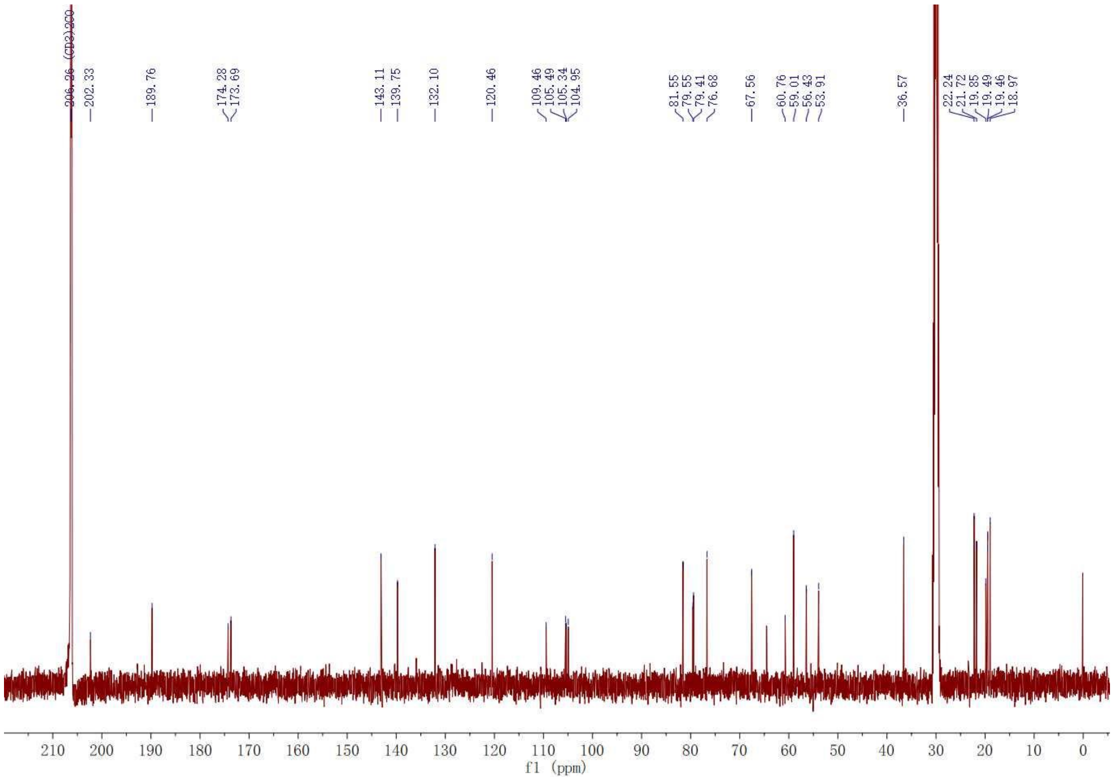

Figure S12. ^{13}C NMR spectrum of **2** (CD_3COCD_3, 125 MHz).

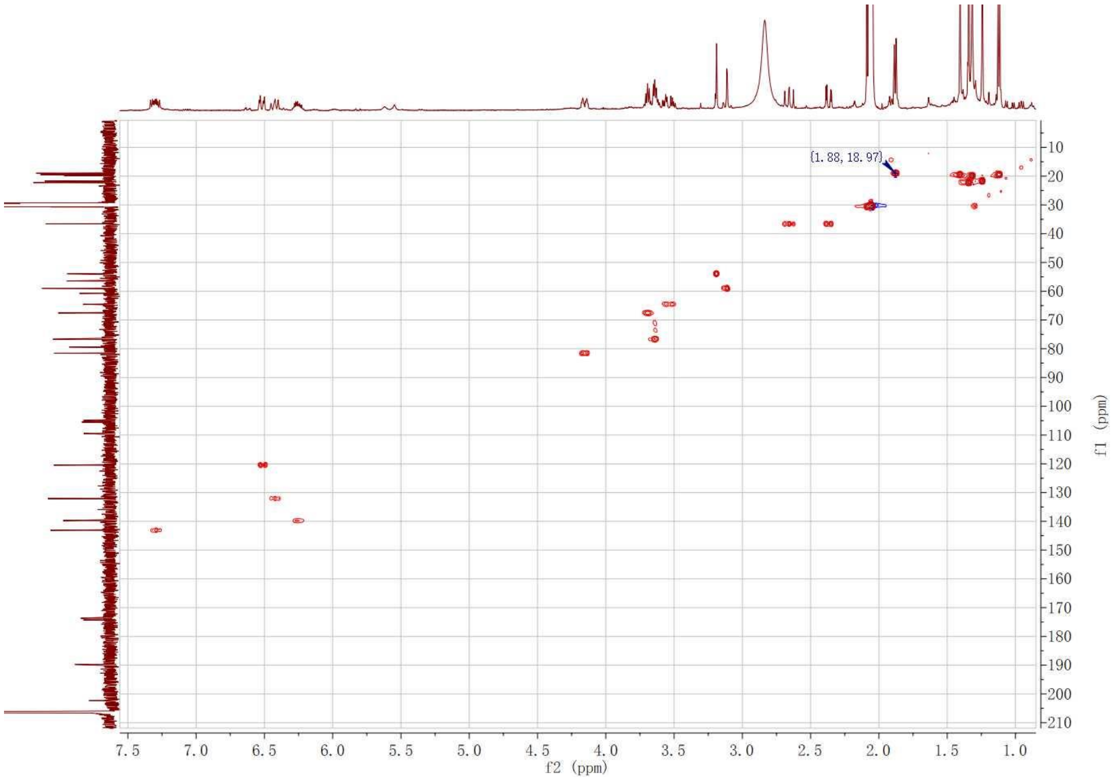

Figure S13. HSQC spectrum of **2**.

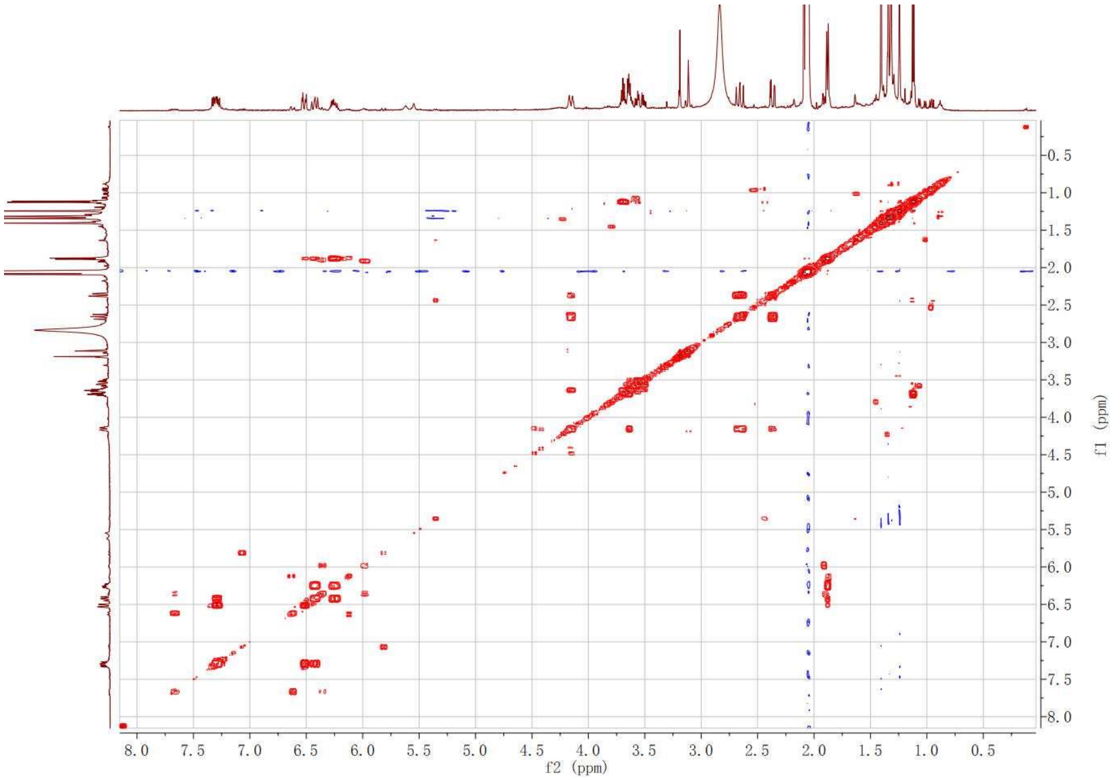

Figure S14. ^{1}H–^{1}H COSY spectrum of **2**.

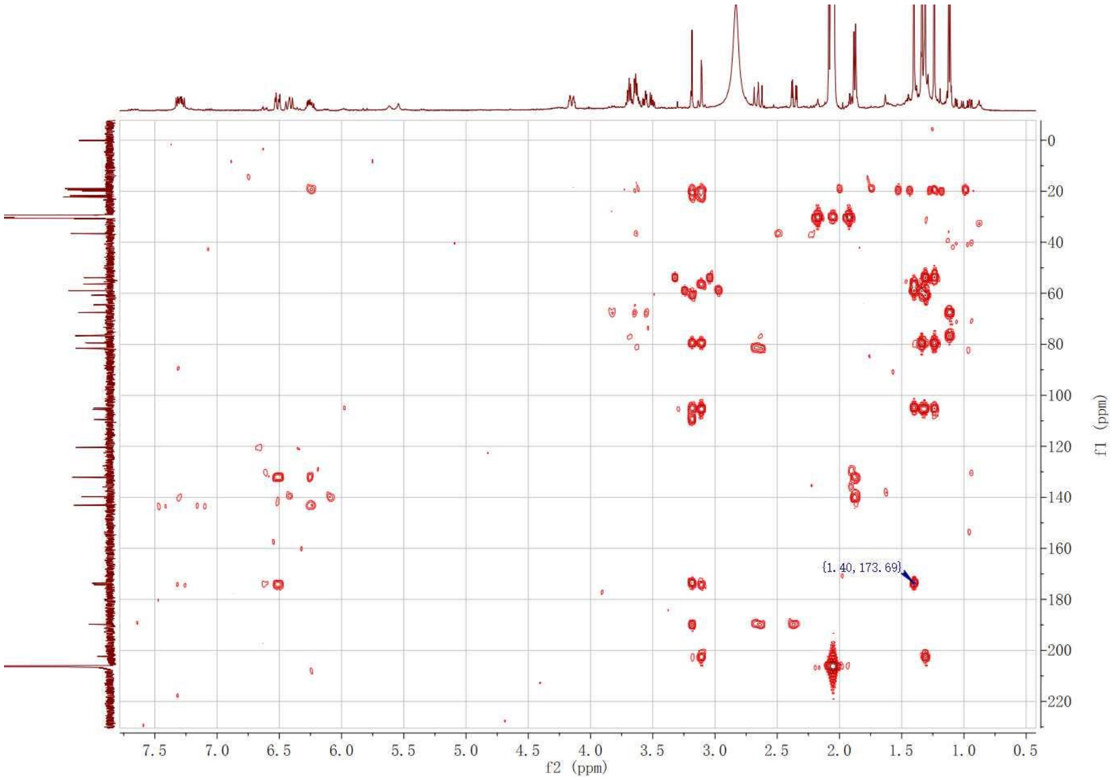

Figure S15. HMBC spectrum of **2**.

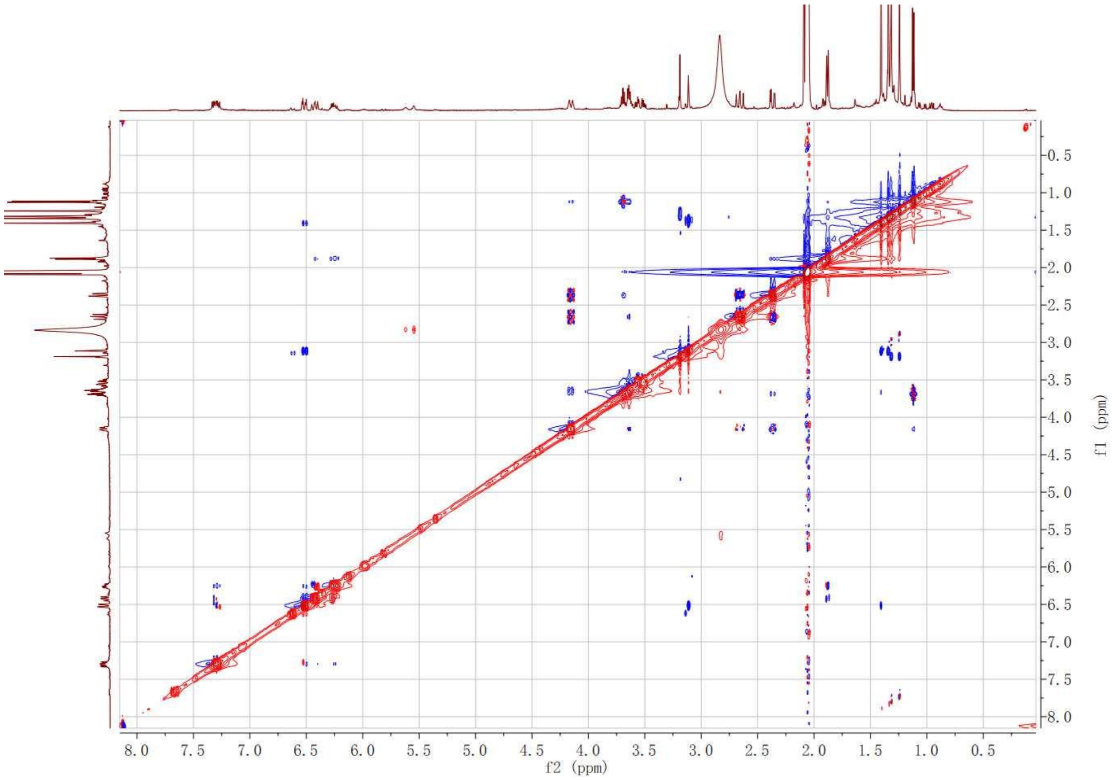

Figure S16. NOESY spectrum of **2**.

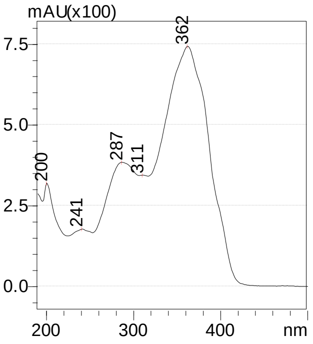

Figure S17. UV spectrum of **2** (100% MeOH/H_2O, extracted from HPLC-DAD data).

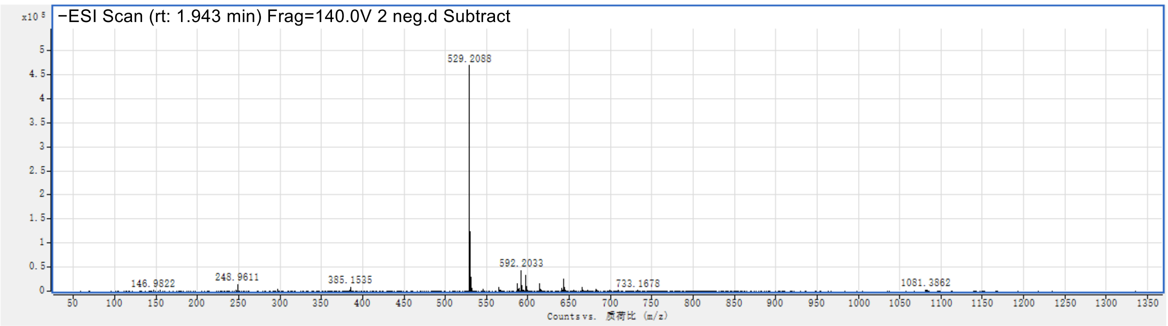

m/z	Calc *m/z*	Diff(ppm)	z	Abund	Formula	Ion
529.2088	529.2079	-1.96	-1	469550	$C_{28}H_{33}O_{10}$	$[M-H]^-$

Figure S18. HRESIMS spectrum of **2**.

Table S1. Cytotoxic activity of compounds **1** and **2**.

Compound	IC_{50} (μg/mL)					
	HCT116	MDA-MB-231	BGC823	Huh-7	PC9	PANC-1
**1**	>24.8	>24.8	>24.8	>24.8	>24.8	>24.8
**2**	>26.5	>26.5	>26.5	>26.5	>26.5	>26.5
CK^+ (Taxol)	0.214	0.257	0.209	0.119	0.326	0.824

Note: Taxol was used as the positive control. DMSO was used to dilute the compounds in the experiments. HCT116, human colon cancer cell line; MDA-MB-231, human breast cancer cell line; BGC823, human gastric cancer cell line; Huh-7, human hepatoma cell line; PC9, human non-small cell lung cancer cell line; PANC-1, human pancreatic cancer cell line.
